# Endothelial stiffening in dyslipidemia

**DOI:** 10.18632/aging.101778

**Published:** 2019-01-22

**Authors:** Elizabeth Le Master, Irena Levitan

**Affiliations:** 1 Division of Pulmonary, Critical Care, Sleep and Allergy, Department of Medicine, University of Illinois at Chicago, Chicago, IL 60607, USA

**Keywords:** endothelial stiffness, oxLDL, CD36, vascular dysfunction

Dyslipidemia, an increase in pro-atherogenic low-density lipoproteins (LDL) and its oxidative modifications, oxLDL, is well known to be a crucial factor in endothelial damage, a key early step and a predictor of the development of atherosclerosis. It is also well-known that atherosclerotic plaques develop in regions of low and non-unidirectional disturbed blood flow as is found by branching points, bifurcations, and in the inner curvature of the aortic arch. Our studies demonstrate a novel paradigm for dyslipidemia-induced endothelial damage, an increase in endothelial stiffness induced by the uptake of oxidized lipids.

Our previous studies showed that exposure to oxLDL in vitro or to plasma dyslipidemia in vivo dramatically increases endothelial stiffness [1–3]. Furthermore, over the last decade, we provided several major insights into the mechanism of oxLDL-induced endothelial stiffening: (i) In contrast to the previous belief, oxLDL does not load aortic endothelial cells with cholesterol, but instead results in incorporation of specific oxysterols, particularly 7-ketocholesterol, and several species of oxidized phospholipids that disrupt lipid packing of the endothelial membrane [1,2]. (ii) OxLDL-induced disruption of lipid packing paradoxically is associated with EC stiffening *via* activation of the contractile RhoA/ROCK cascade, whereas enriching the cells with cholesterol has a rescue effect [3]. (iii) OxLDL-induced stiffening of aortic endothelial cells critically depends on a scavenger receptor CD36 [3,4], known to bind oxLDL and mediate its inflammatory effects [5]. Most recently, we demonstrated that there is a strong synergistic interaction between oxLDL/plasma dyslipidemia and proatherogenic disturbed flow in exacerbating endothelial stiffening via increased expression of CD36 and uptake of oxidized lipids in regions exposed to proatherogenic flow [4]. Specifically, we showed that there is increased stiffening in the endothelial monolayer of the freshly isolated mouse aortic arch as compared to the athero-resistant region of the descending aorta. A short-term high fat diet exasperated endothelial stiffening, an effect attributed to the synergistic interaction of pro-atherogenic disturbed flow and increased uptake of oxidative low-density lipoprotein (oxLDL) [4].

In terms of the functional significance, there is a critical distinction between the *stiffness of the vascular wall*, dominated by extracellular matrix and layers of smooth muscle cells, and *stiffness of the endothelial monolayer*. Previous studies established that dyslipidemia, aging and cardiovascular disease are associated with stiffening of the arterial wall of major arteries, attributed to remodeling of the extracellular matrix and vascular smooth muscles (VSMCs). However, while vascular stiffening is well recognized as an important factor in aging and development of the cardiovascular disease (CVD) [6], the role of endothelial stiffness in vascular dysfunction is only starting to emerge. It is proposed that endothelial stiffening enhances monocyte-endothelial adhesion and exacerbates neovascularization of atherogenic plaques.

Finally, we found that endothelial stiffness increases with age independently from the stiffening of the vascular wall [7]. As was shown previously [8], we found that the stiffness of aortas vascular wall increases with age. Surprisingly, however, endothelial stiffening that develops in parallel to the stiffening of the vascular wall occurred independently and *via* a different mechanism. Specifically, we found that endothelial stiffening but not the stiffening of the vascular wall could be abrogated by the genetic deletion of CD36 or blocking of oxLDL uptake indicating the leading mechanism of endothelial stiffening in aged vessels is accumulating uptake of oxidized lipids. These findings provide a model to discriminate between the roles of endothelial and sub-endothelial stiffness in age-related vascular dysfunction.

In summary, the novel concept of these studies is in discovering the impact of oxidized lipids/plasma dyslipidemia on endothelial stiffness and a new paradigm for synergism between pro-atherogenic flow and dyslipidemia in inducing EC dysfunction: the stiffening of aortic endothelium, mediated by increased expression of CD36 and enhanced oxLDL uptake. Dyslipedimia/aging-induced stiffening of aortic endothelial cells is proposed to constitute an important confounding factor in endothelial dysfunction independent of vascular stiffening.
